# Placement of a self-expandable metallic stent as a bridge to surgery for large bowel obstruction induced by effective neoadjuvant therapy: report of three cases

**DOI:** 10.1186/s40792-018-0509-1

**Published:** 2018-08-23

**Authors:** Fumihiko Ando, Akihisa Matsuda, Masao Miyashita, Satoshi Matsumoto, Nobuyuki Sakurazawa, Youichi Kawano, Hiroshi Yoshida

**Affiliations:** 1 Department of Surgery, Nippon Medical School Chiba Hokusoh Hospital, 1715 Kamagari, Inzai, Chiba 270-1694 Japan; 2 Department of Gastrointestinal and Hepato-Biliary-Pancreatic Surgery, Nippon Medical School, 1-1-5 Sendagi, Bunkyo, Tokyo, 113-8603 Japan

**Keywords:** Obstructive colorectal cancer, Self-expandable metallic colonic stent, Bridge to surgery, Neoadjuvant therapy

## Abstract

**Background:**

Self-expandable metallic stent placement is a widely performed palliative procedure or bridge to surgery for obstructive colorectal cancer. However, the feasibility of this procedure for large bowel obstruction induced by effective neoadjuvant therapy is unclear.

**Case presentation:**

We herein report three such cases involving a 61-year-old man who underwent neoadjuvant chemoradiotherapy for lower rectal cancer, a 56-year-old woman who underwent neoadjuvant chemotherapy for lower rectal cancer, and a 63-year-old woman who underwent neoadjuvant chemotherapy for lower rectal cancer. All were emergently hospitalized with large bowel obstruction that developed while undergoing neoadjuvant therapy. Colonoscopy revealed smooth strictures caused by effective neoadjuvant therapy. Self-expandable metallic stents were placed across the obstruction as a bridge to surgery, and laparoscopic low anterior resection was uneventfully performed in all patients.

**Conclusions:**

We successfully treated three patients with large bowel obstruction induced by a good response to neoadjuvant therapy using self-expandable metallic stents as a bridge to surgery. Further studies with larger sample sizes are warranted to assess the feasibility of this strategy.

## Background

Neoadjuvant therapy (NAT) such as neoadjuvant chemotherapy or neoadjuvant chemoradiotherapy for advanced lower rectal cancer is widely performed to reduce the risk of distant metastasis or local recurrence and to ultimately improve long-term outcomes [1–4]. However, various adverse events may occur in patients undergoing NAT. Effective NAT for advanced rectal cancer occasionally causes a large bowel obstruction (LBO) because of the growth of fibrous and edematous tissue.

Placement of a self-expandable metallic stent (SEMS) to relieve colonic obstruction as a bridge to surgery (BTS) has recently become an effective alternative modality in patients undergoing elective operations for malignant LBO [5]. However, the safety and effectiveness of this BTS approach for secondary cicatricial strictures induced by NAT in patients with colorectal cancer has not been reported.

We encountered three patients with rectal cancer who developed LBO induced by effective NAT and were successfully treated by the BTS approach.

## Case presentation

### Case 1

**Fig. 1:**
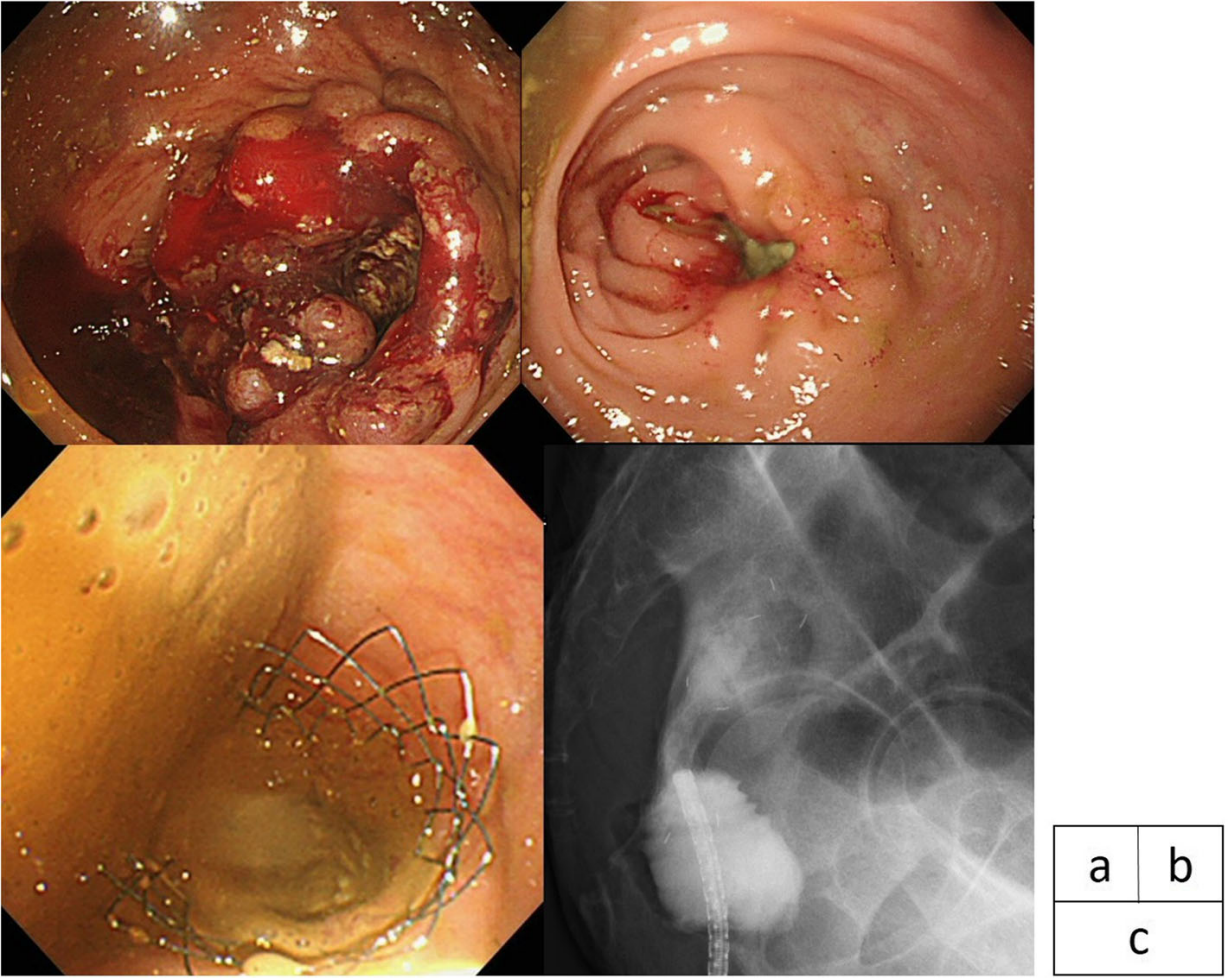
**a** Colonoscopy findings. Colonoscopic examination revealed a circumferential tumor in the lower rectum, 60 mm from the anal verge. **b** Colonoscopy findings. Colonoscopic examination showed smooth stenosis with growth of fibrous tissue that seemed to have been caused by a good response to NAT. **c** Stent placement. A Niti-S Colonic Stent, 8 cm in length by 18 mm in diameter, was placed across the obstruction as a BTS

**Fig. 2:**
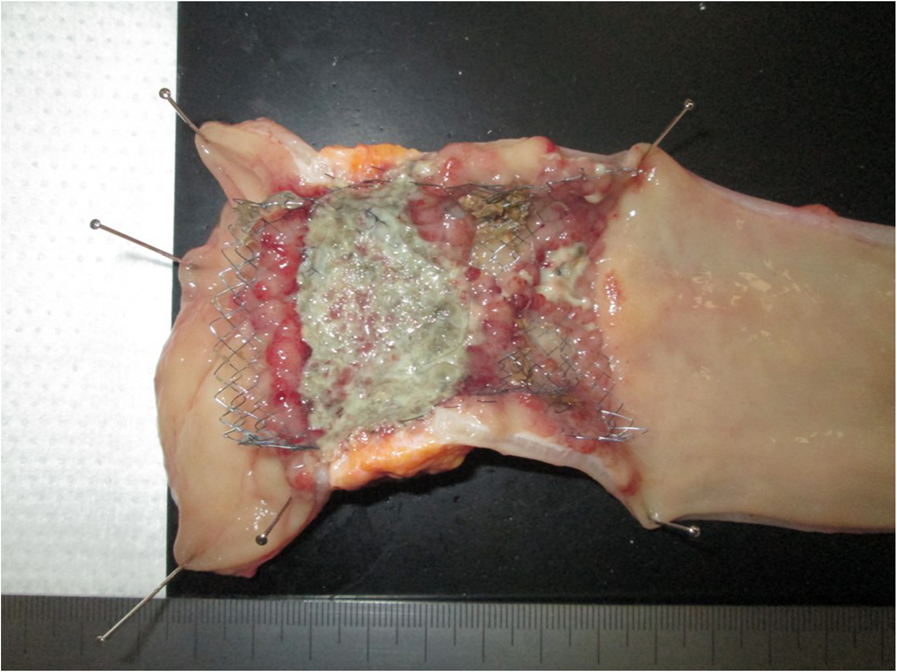
Macroscopic findings. Gross examination of the resected rectal specimen with the stent showed smooth and mild stenosis with growth of fibrous tissue

**Fig. 3:**
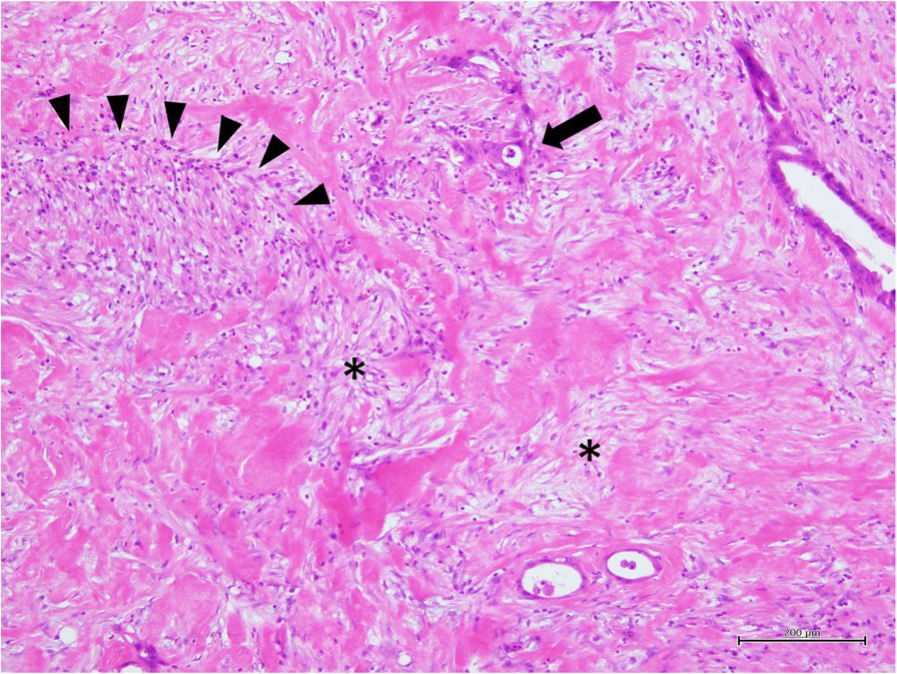
Microscopic findings. Most of the tumor cells were organized by atypical cells (arrow) with growth of fibrous tissue (asterisks) and inflammatory cell infiltration (arrowheads)

A 61-year-old man visited our hospital with constipation. Colonoscopy revealed a circumferential tumor in the lower rectum, 60 mm from the anal verge (Fig. 1a). Biopsy findings indicated a moderately differentiated tubular adenocarcinoma. Although a complete obstruction was not detected, we could not pass the endoscope to the oral side of the tumor. Enhanced computed tomography (CT) demonstrated a 6.3-cm-long bulky middle to lower rectal tumor and multiple enlarged regional lymph nodes without distant metastasis. The patient was diagnosed with cT3N1M0 stage IIIa rectal cancer according to the Japanese Classification of Colorectal Carcinoma 8th edition [6]. Neoadjuvant chemoradiotherapy involving a combination of pelvic radiation (total of 45 Gy for 5 weeks) and concurrent chemotherapy with irinotecan and S-1 was introduced. Three weeks after completion of the therapy, the patient visited our hospital on an emergency basis complaining of no defecation for several days and was diagnosed with LBO based on CT findings. The tumor exhibited a clinical partial response (cPR) to the NAT according to the New Response Evaluation Criteria in Solid Tumors: Revised RECIST Guideline (version 1.1) [7]. Emergency colonoscopy revealed an obstruction at the lower rectum, where the primary tumor was located. Although the tumor had shrunk, we observed smooth stenosis with growth of fibrous tissue, which seemed to have been caused by the good response to NAT (Fig. 1b). A SEMS (Niti-S Colonic Stent; Taewoong Medical Inc., Gimpo-si, Korea) 8 cm in length by 18 mm in diameter was placed across the obstruction as a BTS (Fig. 1c). The patient’s symptoms dramatically improved, and he was discharged uneventfully 3 days after SEMS placement. Laparoscopic low anterior resection with diverting ileostomy was performed 3 weeks after SEMS placement. The duration of the operation was 265 min, and the blood loss volume was very small. The pathological diagnosis was moderately differentiated adenocarcinoma, T3 (SS), INFb, ly1, v2, PN1a, pPM(−), pDM(−), pRM(−), pN0, and stage IIA (Fig. 2). Most of the tumor cells had been replaced by atypical cells with growth of fibrous tissue and inflammatory cell infiltration (Fig. 3). Histopathologically, the chemoradiotherapeutic effect was grade 2. The patient had an uneventful postoperative course and was discharged 14 days after surgery. Capecitabine plus oxaliplatin (XELOX) was started as adjuvant chemotherapy 5 weeks after surgery. At the time of this writing, the patient had been alive without recurrence for 26 months.

### Case 2

**Fig. 4:**
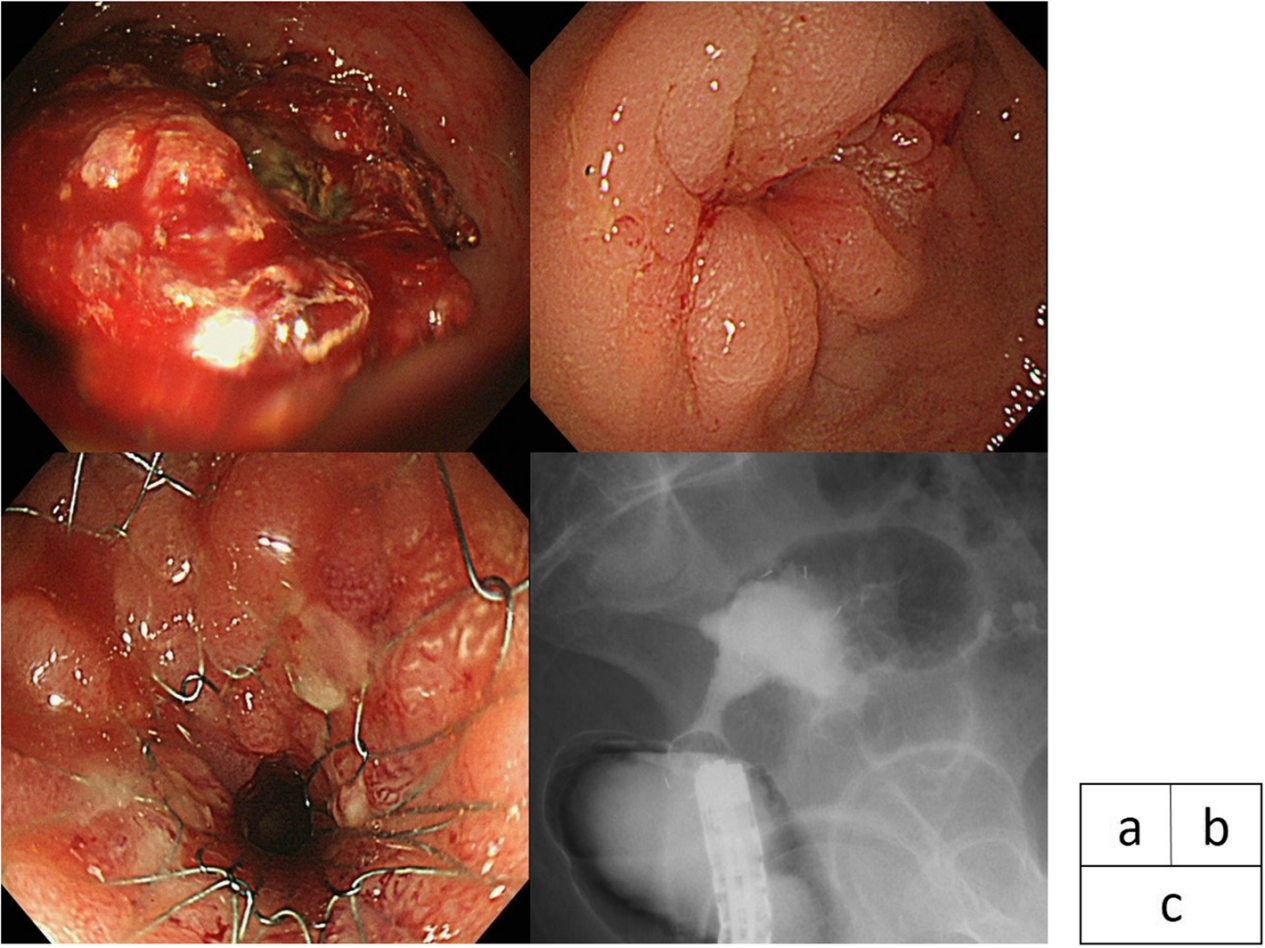
**a** Colonoscopy findings. Colonoscopic examination revealed a circumferential tumor in the lower rectum, 45 mm from the anal verge. **b** Colonoscopy findings. The shape of the stenosis was smooth and edematous that seemed to have been caused by a good response to NAT. **c**. Stent placement. A Niti-S Colonic Stent, 6 cm in length by 18 mm in diameter, was placed across the obstruction as a BTS

A 56-year-old woman presented because of lack of defecation. She underwent colonoscopy, and a circumferential tumor was found in the lower rectum, 45 mm from the anal verge (Fig. 4a). The tumor was diagnosed as cT4bN2M0 stage IIIb rectal cancer. XELOX plus bevacizumab was introduced as NAT. Upon completion of five courses, the patient underwent colonoscopy for evaluation of the response to NAT. Circumferential luminal narrowing was found in the lower rectum, where the primary tumor was located. The shape of the stenosis was smooth and edematous (Fig. 4b). CT findings revealed LBO. The tumor exhibited a cPR to the NAT. We estimated that the stenosis was associated with effective NAT, as in case 1. A SEMS (Niti-S Colonic Stent) 6 cm in length by 18 mm in diameter was placed across the stenosis as a BTS (Fig. 4c). Laparoscopic low anterior resection with diverting ileostomy was performed 6 weeks after SEMS placement. The duration of the operation was 308 min, and the blood loss volume was very small. The pathological diagnosis was moderately differentiated adenocarcinoma, T3 (SS), INFb, ly1, v1, PN0, pPM(−), pDM(−), pRM(−), pN0, and stage IIA. Most of the tumor cells were organized by atypical cells with growth of fibrous tissue and inflammatory cell infiltration. Histopathologically, the chemotherapeutic effect was grade 2. The patient had an uneventful postoperative course and was discharged 20 days after surgery. XELOX was started as adjuvant chemotherapy 5 weeks after surgery. At the time of this writing, the patient had been alive without recurrence for 17 months.

### Case 3

A 63-year-old woman presented with bloody stool. Colonoscopy revealed a circumferential tumor in the lower rectum, 80 mm from the anal verge. The tumor was diagnosed as cT3N2M0 stage IIIb rectal cancer. mFOLFOX6 plus cetuximab was started as NAT. Upon completion of five courses, the patient visited our hospital on an emergency basis complaining of no defecation for several days. Emergency colonoscopy showed a stenosis in the lower rectum, where the primary tumor was located. CT showed that the tumor had obviously shrunk and that an LBO had developed. The tumor exhibited a cPR to the NAT. We estimated that the stenosis had been caused by effective NAT, as in cases 1 and 2. A SEMS (Niti-S Colonic Stent) 6 cm in length by 18 mm in diameter was placed as a BTS across the stenosis. After SEMS placement, the patient began oral intake and NAT was restarted immediately. Upon completion of six courses, laparoscopic low anterior resection with diverting ileostomy was performed. The duration of the operation was 218 min, and the blood loss volume was very small. The pathological diagnosis was well-differentiated adenocarcinoma, T3 (SS), INFc, ly0, v1, PN1a, pPM(−), pDM(−), pRM(−), pN1 (1/18), and stage IIIa. The tumor cells contained atypical cells with growth of fibrous tissue and inflammatory cell infiltration. Histopathologically, the chemotherapeutic effect was grade 2. The patient had an uneventful postoperative course and was discharged 24 days after surgery. mFOLFOX6 was started after surgery as adjuvant chemotherapy. At the time of this writing, the patient had been alive without recurrence for 11 months.

## Discussion

LBO with growth of fibrous and edematous tissue can occur as an adverse event during NAT. Neoadjuvant chemoradiotherapy for rectal cancer causes tumor regression by eradication and replacement of carcinoma cells by fibrous or fibroinflammatory tissues [8]. Previous studies have shown that patients with fibroinflammatory changes after neoadjuvant chemoradiotherapy have better disease-free survival [9, 10]. The cicatricial strictures found in our patients were considered to be a result of these fibroinflammatory changes after effective NAT. The number of patients who develop LBO induced by effective NAT for lower rectal cancer is expected to increase because of recent developments in NAT [11]. Actually, a total of 23 patients with rectal cancer underwent NAT (neoadjuvant chemotherapy, 10 patients; neoadjuvant chemoradiotherapy, 13 patients) at our single institution from January 2012 to March 2017. Of these, the three patients (13.0%) in the present report developed an LBO associated with effective NAT.

The three cases described herein suggest that SEMS placement can be a good strategy as a BTS for LBO associated with a good response to NAT. In general, three treatment options are available for LBO: an emergency operation, transanal decompression tube placement, and SEMS placement. SEMS placement as a BTS is a widely accepted alternative intervention for malignant LBO because its short-term outcomes and preoperative quality of life are better than those of emergency surgery and transanal decompression tube placement [12, 13]. Preoperative worse nutritional and immunological status is well known to be a risk of postoperative complications after colorectal cancer surgery [14, 15]. Therefore, a plausible contributing factor for better short-term outcomes of BTS strategy using SEMS is a quick and sufficient preoperative recovery of general and intestinal conditions after SEMS placement compared with other treatment choices [13]. However, the feasibility and effectiveness of SEMS placement for LBO induced by effective NAT has not been evaluated. Incidentally, for patients with esophageal cancer who develop stenosis after radiotherapy and chemotherapy, stent therapy is clinically recommended to achieve a return to oral intake according to the Japanese guidelines for diagnosis and treatment of esophageal carcinoma [16]. A previous study showed that polyflex stent placement to treat malignant dysphagia in patients with locally advanced esophageal cancer during NAT such as neoadjuvant chemoradiotherapy and neoadjuvant chemotherapy is safe and allows oral feeding [17].

The selection of the most appropriate SEMS with respect to the type and diameter for LBO induced by effective NAT is a critical concern for successful placement. In our institution, we usually select the 18-mm-diameter Niti-S Colonic Stent, the radial force of which is mild considering the vulnerability of the rectal wall when affected by NAT-induced fibroinflammation.

Generally, SEMS placement as a BTS for lower rectal cancer has not been recommended because of problems such as anal pain, anastomotic leakage, and difficulty in anal preservation. When placing a SEMS for lower rectal cancer, we believe that the distance between the proximal edge of the SEMS and the anal edge of the tumor should be kept at ≤ 10 mm. Although it is generally thought to be difficult to place the proximal edge of a distal-releasing type SEMS exactly at the most appropriate location, the stents in the current cases were successfully placed by the expert endoscopist in our institution. Placement of an uncovered stent with a proximal-releasing delivery system is reportedly a feasible, safe, and effective treatment for patients with a malignant lower rectal obstruction within 5 cm from the anal verge [18]. However, proximal-releasing delivery systems are not available in Japan. Furthermore, the surgeon should be careful to avoid involving a stent at the time of rectal resection. We always use colonoscopy to ensure that a stent is not involved just before resection of the rectum.

Although the short-term safety and efficacy of SEMS placement as a BTS for malignant LBO are well known, the long-term oncologic effects of SEMS placement for LBO remain unclear. According to the 2015 European Society of Gastrointestinal Endoscopy Clinical Guideline [19], SEMS placement as a BTS in patients undergoing elective procedures is not recommended as a standard treatment of symptomatic left-sided malignant LBO because previous studies have shown high perforation rates and inferiority in long-term outcomes of stent therapy when compared with emergency surgery. However, our recent meta-analysis of 1136 patients from 11 studies demonstrated that a BTS strategy showed non-inferiority in long-term outcomes when compared with emergency surgery [20].

## Conclusions

We encountered three patients with LBO caused by a good response to NAT who were successfully treated by SEMS placement as a BTS. Further studies with larger sample sizes are needed to fully evaluate the safety and efficacy of this technique.

## Abbreviations

BTS: Bridge to surgery
cPR: Clinical partial response
CT: Computed tomography
LBO: Large bowel obstruction
NAT: Neoadjuvant therapy
SEMS: Self-expandable metallic stent
